# The association of childhood trauma with depressive and negative symptoms in recent onset psychosis: a sex-specific analysis

**DOI:** 10.1017/S0033291723001824

**Published:** 2023-12

**Authors:** Anne-Sophie D. Enthoven, Shiral S. Gangadin, Lieuwe de Haan, Wim Veling, Erik F. J. de Vries, Janine Doorduin, HAMLETT and OPHELIA Consortium, Marieke J. H. Begemann, Iris E. C. Sommer

**Affiliations:** 1 Department of Nuclear Medicine and Molecular Imaging, University of Groningen, University Medical Center Groningen, Groningen, The Netherlands; 2 Department of Biomedical Sciences of Cells and Systems, and Department of Psychiatry, University of Groningen, University Medical Center Groningen, Groningen, The Netherlands; 3 Department of Early Psychosis, Amsterdam UMC, Academic Medical Center, Amsterdam, The Netherlands; 4 Department of Psychiatry, University of Groningen, University Medical Center Groningen, Groningen, The Netherlands

**Keywords:** Childhood trauma, depressive symptoms, first-episode psychosis, negative symptoms, psychosis, schizophrenia, sex

## Abstract

**Background:**

Childhood trauma may impact the course of schizophrenia spectrum disorders (SSD), specifically in relation to the increased severity of depressive or negative symptoms. The type and impact of trauma may differ between sexes. In a large sample of recent-onset patients, we investigated the associations of depressive and negative symptoms with childhood trauma and whether these are sex-specific.

**Methods:**

A total of 187 first-episode psychosis patients in remission (Handling Antipsychotic Medication: Long-term Evaluation of Targeted Treatment study) and 115 recent-onset SSD patients (Simvastatin study) were included in this cross-sectional study (men: *n* = 218; women: *n* = 84). Total trauma score and trauma subtypes were assessed using the Childhood Trauma Questionnaire Short Form; depressive and negative symptoms were rated using the Positive And Negative Symptoms Scale. Sex-specific regression analyses were performed.

**Results:**

Women reported higher rates of sexual abuse than men (23.5% *v.* 7.8%). Depressive symptoms were associated with total trauma scores and emotional abuse ratings in men (*β*: 0.219–0.295; *p* ≤ 0.001). In women, depressive symptoms were associated with sexual abuse ratings (*β*: 0.271; *p* = 0.011). Negative symptoms were associated with total trauma score and emotional neglect ratings in men (*β*: 0.166–0.232; *p* ≤ 0.001). Negative symptoms in women were not linked to childhood trauma, potentially due to lack of statistical power.

**Conclusions:**

Depressive symptom severity was associated with different types of trauma in men and women with recent-onset SSD. Specifically, in women, depressive symptom severity was associated with childhood sexual abuse, which was reported three times as often as in men. Our results emphasize the importance of sex-specific analyses in SSD research.

## Introduction

Childhood trauma is a well-known risk factor for the development of schizophrenia spectrum disorders (SSD) (Gibson, Alloy, & Ellman, 2016; Varese et al., 2012). Childhood trauma comprises different subtypes, including sexual, physical, and emotional abuse, as well as physical and emotional neglect. Recent work suggests that differences in the clinical presentation of SSD are linked to the presence or absence of a traumatic history (Alameda et al., 2021; Popovic et al., 2019). Trauma-exposed patients experience more severe positive, negative, and depressive symptoms, worse cognitive performance, more treatment resistance, and decreased global functioning (Alameda et al., 2021; Bailey et al., 2018; Gibson et al., 2016; Vargas et al., 2019; Vila-Badia et al., 2021).

Female SSD patients report a higher exposure to childhood trauma than male SSD patients (Comacchio, Lasalvia, & Ruggeri, 2019a; Fernando, Sommer, & Hasan, 2020) and the specific types of trauma also differ. Overall, women experience more sexual abuse than men, both in first-episode psychosis and chronic patients (Comacchio et al., 2019a; Vila-Badia et al., 2021). Moreover, emotional abuse and physical neglect are also more common in women with SSD, while emotional neglect is more common in men (Vila-Badia et al., 2021). In addition to sex differences in the type of trauma exposure, women and men may respond differently to trauma (Stanton, Denietolis, Goodwin, & Dvir, 2020). Sex-specific coping styles can impact the risk for developing certain psychiatric symptoms later in life (Stanton et al., 2020). In SSD, the severity of childhood trauma has been consistently associated with more severe depressive and negative symptoms (Alameda et al., 2021), yet evidence on the potentially moderating effect of sex remains inconclusive. Pruessner et al. (2019) reported that childhood trauma was mainly related to mood symptoms in women and to negative symptom severity in men. These findings are corroborated by a recent review by Comacchio et al. (2019a). However, other studies, including an original investigation from the same group, did not find any sex- differences in the association between trauma and depressive and negative symptoms (Comacchio et al., 2019b; Ruby, Rothman, Corcoran, Goetz, & Malaspina, 2017). Another study reported emotional neglect and physical abuse to be related to more severe depressive and negative symptoms specifically in women but not in men (Garcia et al., 2016; Kelly et al., 2016). Discrepancies in these previous findings may be partly explained by the fact that most studies have investigated participants with established SSD rather than a recent-onset population, without correcting for possible confounders such as illness duration and chronic medication use (Wang et al., 2013). In addition, the majority of studies investigated childhood trauma only as a binary factor, while others evaluated trauma as a continuous variable of interest, allowing to account for the severity of childhood trauma exposure.

So far, only a few studies investigated sex-specificity in trauma-related symptomatology in first-episode psychosis and recent-onset patients (Comacchio et al., 2019b; Garcia et al., 2016; Pruessner et al., 2019), including relatively small sample sizes (i.e. *n* = 79 and *n* = 212) and/or investigating only a few types of childhood trauma (i.e. only physical and sexual abuse). However, sex differences in the incidence and impact of childhood trauma may importantly contribute to the observed sex differences in the development and course of SSD. On average, men show a higher risk to develop SSD, have an earlier age of onset, have lower social and professional functioning at onset, have a higher relapse rate, and show a worse response to antipsychotic medication, although findings are age-dependent (Fernando et al., 2020; Franceschini & Fattore, 2021; Merikangas & Almasy, 2020; Sommer, Tiihonen, van Mourik, Tanskanen, & Taipale, 2020). Sex differences are also found with respect to symptom domains, with more severe affective symptoms in women and more severe negative symptoms and cognitive deficits in men (Fernando et al., 2020; Franceschini & Fattore, 2021; Merikangas & Almasy, 2020; Sommer et al., 2020). Sex differences in the severity of positive symptoms are much less pronounced (Franceschini & Fattore, 2021).

We conducted a large study to investigate sex differences in the prevalence of a wide range of trauma subtypes, including emotional abuse, emotional neglect, physical abuse, physical neglect, and sexual abuse and to assess the association of these childhood trauma subtypes with depressive and negative symptoms in first-episode psychosis and recent-onset SSD patients. We hypothesize that childhood trauma is associated with predominantly depressive symptoms in women and with mainly negative symptoms in men.

## Methods

### Participants

Data were obtained from the baseline measurements of both the Simvastatin augmentation for recent-onset psychotic disorder (Simvastatin) study (Begemann et al., 2015; Sommer et al., 2021) and the ongoing Handling Antipsychotic Medication: Long-term Evaluation of Targeted Treatment (HAMLETT) study (Begemann et al., 2020). All participants signed informed consent. Procedures were performed in accordance with the declaration of Helsinki (64th WMA general assembly; October 2013). The Simvastatin study was approved by the institutional review board of the University Medical Center Utrecht (UMCU) (Clinicaltrials.gov: NCT01999309) and the HAMLETT study was approved by the research and ethics committee of the University Medical Center Groningen (UMCG) (EudraCT number: 2017-002406-12).

The study protocols of the respective studies are described in more detail in Begemann et al. (2015) and Begemann et al. (2020). For the Simvastatin study, patients were recruited from Dutch in- and outpatient settings between November 2013 and February 2019. Patients of 18–50 years old with a diagnosis of schizophrenia, schizoaffective, schizophreniform disorder or unspecified schizophrenia spectrum and other psychotic disorders were eligible. The first SSD diagnosis was within three years before the patient was included in the study and all patients were in remission at the moment of inclusion. For the ongoing HAMLETT study, patient recruitment started in September 2017 and takes place in outpatient clinics of 25 Dutch mental healthcare centers. We included patients who started their participation before October 2021. Patients of 16–60 years old who were in remission for 3–6 months of their firstepisode of schizophrenia, schizoaffective disorder, schizophreniform disorder, brief psychotic disorder, delusional disorder, substance/medication-induced psychotic disorder, or unspecified schizophrenia spectrum and other psychotic disorders were included.

In both studies, the Comprehensive Assessment of Symptoms and History (CASH) (Andreasen, Flaum, & Arndt, 1992) was used to confirm the diagnoses and illness duration as established by the treating psychiatrist. Antipsychotic use was recorded and used dosage was converted to chlorpromazine equivalent dose (Gardner, Murphy, O'Donnell, Centorrino, & Baldessarini, 2010; Leucht, Samara, Heres, & Davis, 2016; Leucht et al., 2014).

### Procedures

The Positive And Negative Symptom Scale (PANSS) was used to assess depressive and negative symptoms. Depressive symptoms were measured using category ‘Depressed’ from the PANSS five-factor model, which includes the single items G2 (anxiety), G3 (guilt feeling), and G6 (depression posturing) (Wallwork, Fortgang, Hashimoto, Weinberger, & Dickinson, 2012). The PANSS negative subscale was used to assess negative symptoms.

Childhood trauma was assessed using the Dutch version of the Childhood Trauma Questionnaire – Short Form (CTQ-SF) (Bernstein et al., 2003; Thombs, Bernstein, Lobbestael, & Arntz, 2009). The CTQ-SF assesses five specific forms of trauma (emotional, physical, and sexual abuse and emotional and physical neglect) by asking participants to rate 25 propositions (i.e. five per subscale) concerning trauma on a scale of 1–5 with 1 denoting no exposure and 5 denoting a very high exposure to the trauma described in the proposition. For example, one of the propositions of the subscale of physical neglect is ‘during my childhood, my parents were too drunk or stoned to care for the family’. The score-range per subscale is 5–25. These sub-scores can be added up to yield a total trauma score (range: 25–125). For each trauma subtype, patients were categorized as trauma-exposed based on the established CTQ cut-off scores for moderate to severe trauma exposure, which were adopted from the CTQ manual (emotional abuse ≥13; emotional neglect ≥15; physical abuse ≥10; physical neglect ≥10; sexual abuse ≥8) (Bernstein et al., 2003). The cut-off scores were used to assess the prevalence of the trauma subtypes. Prevalence of total trauma was assessed by defining total trauma as having at least 1 subtype of trauma (Bernstein et al., 2003). The continuous measures of the total trauma and trauma subtypes were used for the regression analyses.

### Statistical analyses

Statistical analyses were performed using IBM Statistical Package for Social Sciences (SPSS) version 23. Mann–Whitney *U* tests were used to assess differences between men and women in demographic and clinical variables. Fisher's exact test was used to examine sex differences in trauma prevalence. Linear regression analyses were performed to assess the main effect of trauma on two separate symptom domains (i.e. depressive and negative symptoms), first in the total sample and subsequently in men and women separately. The effects of both the total trauma score and the trauma sub-scores were assessed. We verified that the assumptions of linear regression were met; scores were not log-transformed. We corrected the regression analyses for covariates (i.e. age and chlorpromazine equivalent dose for all analyses, and sex for the analyses of the total population). Positive symptoms were also not used as covariates because of the low scores and in order to enable comparison with other studies, which also do not use symptom scores as covariates. Results were presented as standardized beta (*β*), *t*-statistic, *R*^2^ and *p* value. *R*^2^ denotes the unique variance explained by the independent variable after correction for the covariates; it is determined using the linear regression model with the covariates in the first block and the addition of the independent variable (i.e. the trauma subtype) in the second block.

Both for the Mann–Whitney *U* tests and for the regression analyses, we corrected for multiple testing using the Benjamini–Hochberg correction (Benjamini & Hochberg, 1995), a step-wise procedure for correction for the False Discovery Rate (FDR) in which the *p* value of finding *i* is considered to be significant if the original *p* value *p*(*i*) is lower than the corrected *p* value calculated using the formula *p*(*i*) < *Q* × (*R*(*i*)/*n*), in which *R*(*i*) is the rank of the *p* value, *n* is the number of tests, and *Q* is the FDR, which was set on 0.05. The number of regression analyses performed was 36, since we tested six trauma scales for two symptom domains, and we performed these analyses for the total sample, men, and women.

## Results

### Participants

In total, 302 patients [Simvastatin study, *n* = 115 (38.1%); HAMLETT, *n* = 187 (61.9%)] completed both the CTQ-SF and the PANSS measurements. Table 1 shows the mean ± standard deviation (s.d.) of the baseline demographic characteristics, the CTQ-SF trauma ratings, and the PANSS depressive and negative ratings. The baseline demographic characteristics of the recent-onset patients (i.e. participants of the Simvastatin study) and the first-episode psychosis patients (i.e. participants of the HAMLETT study) are shown separately in online Supplementary Table S1. The recent-onset subgroup had a shorter duration of illness and were treated with lower chlorpromazine equivalents, lower negative and depressive symptoms, lower total trauma scores, and lower emotional abuse scores.

**Table 1.** Demographic and clinical characteristics

	All participants (*n* = 302)		Men (*n* = 218)		Women (*n* = 84)		*p* value Mann–Whitney test	*p* value Fisher's exact test
Male [*n* (%)]	218 (72.2%)		N.A.		N.A.			
Age (years)	28.3 (8.1)		27.7 (7.7)		29.8 (9.1)		0.096	
Illness duration (months)	12.2 (13.5)		12.8 (15.0)		10.7 (8.3)		0.550	
Chlorpromazine equivalent dose (mg/day)	255 (184)		273 (194)		209 (148)		0.015*	
PANSS								
Negative symptom subscale PANSS	12.9 (4.8)		13.2 (4.9)		12.2 (4.6)		0.089	
Depressive symptoms (PANSS 5-factor model)	6.4 (2.8)		6.3 (2.8)		6.6 (2.7)		0.281	
CTQ-SF								
Total trauma score	39.4 (11.3)	37.1%	38.6 (10.0)	34.4%	41.7 (13.9)	44.0%	0.345	0.078
Emotional abuse	8.5 (3.7)	13.8%	8.2 (3.4)	11.0%	9.3 (4.5)	21.2%	0.275	0.025*
Emotional neglect	11.6 (4.3)	23.4%	11.5 (4.0)	21.6%	11.8 (4.9)	28.2%	0.948	0.226
Physical abuse	6.0 (2.2)	5.6%	5.9 (2.1)	5.5%	6.3 (2.4)	5.9%	0.165	1.000
Physical neglect	7.2 (2.5)	12.5%	7.2 (2.4)	11.9%	7.2 (2.7)	14.1%	0.734	0.566
Sexual abuse	6.1 (3.2)	12.2%	5.7 (2.6)	7.8%	7.2 (4.1)	23.5%	<0.001**	<0.001*

Data are represented as mean (s.d.). *p* values denote the differences between men and women. For childhood trauma scores, *p* values for both continuous measures and prevalence are shown. * *p* < 0.05. ** Significant after Benjamini–Hochberg FDR correction. The FDR-derived significance threshold was 0.005. No FDR correction was applied for Fisher's exact test.

### Sex differences in demographic and clinical characteristics

When evaluating childhood trauma scores as a continuous measure, women reported higher scores of sexual abuse than men (men: 5.7 ± 2.6; women: 7.2 ± 4.1; *p* < 0.001), but no significant sex differences were observed for other trauma subtypes (*p* > 0.05). When examining childhood trauma prevalence (i.e. using the dichotomous measure), we found that sexual abuse rates were three times higher in women as compared to men (23.5% *v.* 7.8%; *p* < 0.001). The prevalence of emotional abuse was twice as high in women relative to men (21.2% *v.* 11.0%; *p* = 0.025). The PANSS depressive scores did not differ significantly between the groups (men: 6.3 ± 2.8; women: 6.6 ± 2.7; *p* = 0.281). The PANSS negative scores were not significantly different between the sexes either (*p* = 0.089), although men (13.2 ± 4.9) scored marginally higher than women (12.2 ± 4.6). Men used a higher chlorpromazine equivalent dose (273 ± 194) than women (209 ± 148; *p* = 0.015), but the difference was not statistically significant anymore after FDR correction.

### Depressive symptoms

**Figure 1.**
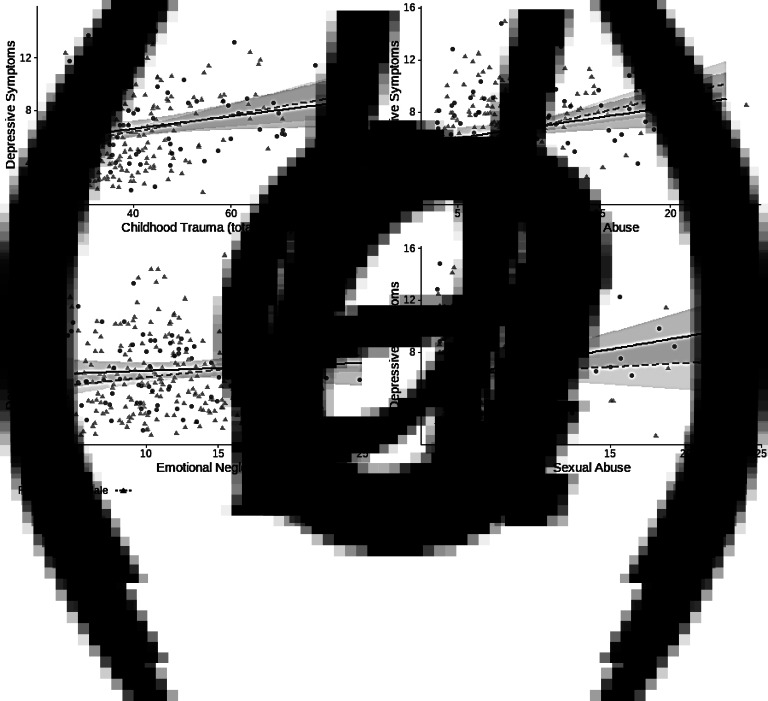
Regression coefficients of the association between depressive symptoms and (*a*) total trauma score, (*b*) emotional abuse, (*c*) emotional neglect, and (*d*) sexual abuse, separately for men and women. The link between both total trauma and emotional abuse on depressive symptoms was significant in the total sample and in men. Trend-level effects were observed for women. The association between emotional neglect and depressive symptoms in the total sample and in men did not survive correction for multiple comparisons. Sexual abuse was significantly associated with depressive symptoms in women.

In the sample with men and women combined, more severe depressive symptoms were significantly associated with higher total trauma scores (*R*^2^: 0.050; *β*: 0.226; *p* < 0.001; Table 2; Fig. 1*a*). Specifically, emotional abuse (*R*^2^: 0.077; *β*: 0.280; *p* < 0.001; Fig. 1*b*) showed an even stronger association with depressive symptoms, while emotional neglect (*R*^2^: 0.021; *β*: 0.135; *p* = 0.021; Table 2; Fig. 1*c*) and sexual abuse (*R*^2^: 0.019; *β*: 0.140; *p* = 0.019; Table 2; Fig. 1*d*) only reached trend-level significance after correcting for multiple comparisons.

**Table 2.** Regression coefficients of the associations between childhood trauma and depressive symptoms with correction for covariates (i.e. age and chlorpromazine equivalent dose for all analyses, and sex for the analyses of the total population)

	All participants			Men			Women		
	*R*^2^ change	*β*	*p* value	*R*^2^ change	*β*	*p* value	*R*^2^ change	*β*	*p* value
Total trauma score	0.050	0.226	<0.001**	0.047	0.219	0.001**	0.050	0.224	0.036*
Emotional abuse	0.077	0.280	<0.001**	0.085	0.295	<0.001**	0.061	0.247	0.020*
Emotional neglect	0.018	0.135	0.021*	0.024	0.156	0.025*	0.005	0.069	0.523
Physical abuse	0.010	0.099	0.092	0.009	0.096	0.165	0.005	0.072	0.512
Physical neglect	0.011	0.107	0.068	0.010	0.098	0.156	0.019	0.137	0.203
Sexual abuse	0.019	0.140	0.018*	0.003	0.053	0.442	0.072	0.271	0.011**

* *p* < 0.05. ** Significant after Benjamini–Hochberg FDR correction. The FDR-derived significance threshold was 0.014.

Results for men separately were almost similar to the results of men and women combined, as associations between depressive symptoms and total trauma score (*R*^2^: 0.047; *β*: 0.219; *p* = 0.001; Fig. 1*a*) and emotional abuse (*R*^2^: 0.085; *β*: 0.295; *p* < 0.001; Fig. 1*b*) were significant, while the association with emotional neglect did not survive multiple comparison correction (*R*^2^: 0.024; *β*: 0.156; *p* = 0.025; Fig. 1*c*).

In women, sexual abuse was significantly associated with increased severity of depressive symptoms (*R*^2^: 0.072; *β*: 0.271; *p* = 0.011; Fig. 1*d*). Trend-level effects were observed for depressive symptoms with total trauma score (*R*^2^: 0.050; *β*: 0.224; *p* = 0.036; Fig. 1*a*) and emotional abuse severity (*R*^2^: 0.020; *β*: 0.247; *p* = 0.020; Fig. 1*b*).

### Negative symptoms

For the total sample, higher total trauma scores were significantly associated with more severe negative symptoms (*R*^2^: 0.027; *β*: 0.166; *p* = 0.003). Among the trauma subtypes, both emotional abuse (*R*^2^: 0.019; *β*: 0.140; *p* = 0.013) and emotional neglect ratings (*R*^2^: 0.038; *β*: 0.195; *p* < 0.001) were significantly associated with negative symptoms (Table 3).

**Table 3.** Regression coefficients of the associations between childhood trauma and negative symptoms with correction for covariates (i.e. age and chlorpromazine equivalent dose for all analyses, and sex for the analyses of the total population)

	All participants			Men			Women		
	*R*^2^ change	*β*	*p* value	*R*^2^ change	*β*	*p* value	*R*^2^ change	*β*	*p* value
Total trauma score	0.027	0.166	0.003**	0.027	0.166	0.011**	0.028	0.167	0.126
Emotional abuse	0.019	0.140	0.013**	0.018	0.135	0.041*	0.019	0.140	0.202
Emotional neglect	0.038	0.195	<0.001**	0.053	0.232	<0.001**	0.014	0.117	0.285
Physical abuse	0.012	0.109	0.051	0.010	0.099	0.130	0.018	0.137	0.214
Physical neglect	0.010	0.100	0.074	0.006	0.080	0.225	0.022	0.148	0.174
Sexual abuse	<0.001	0.006	0.912	0.002	−0.042	0.522	0.009	0.093	0.397

* *p* < 0.05. ** Significant after Benjamini–Hochberg FDR correction. The FDR-derived significance threshold was 0.014.

**Figure 2.**
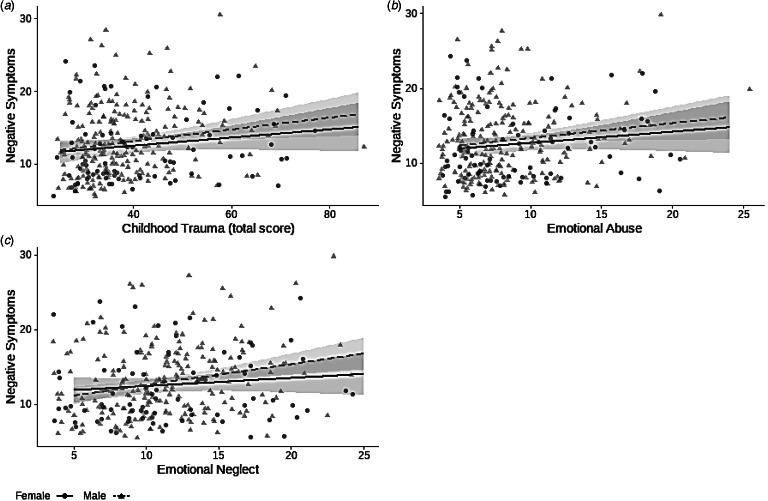
Regression coefficients of the effect of (*a*) total trauma score, (*b*) emotional abuse, and (*c*) emotional neglect on negative symptoms, separately for men and women. These relations were significant in the total sample. Both total trauma score and emotional neglect were significantly associated with negative symptoms in men. For emotional neglect, the effect on negative symptoms in men was no longer significant when comparing multiple comparisons. In women, there were no significant relationships between any form of childhood trauma and negative symptoms.

In the sex-specific analyses, both total trauma scores (*R*^2^: 0.027; *β*: 0.166; *p* = 0.011) and emotional neglect ratings (*R*^2^: 0.053; *β*: 0.232; *p* < 0.001) were significantly associated with negative symptoms in men (Fig. 2*a*, *b*). The relation between negative symptoms and emotional abuse in men did not survive FDR correction (*R*^2^: 0.018; *β*: 0.135; *p* = 0.041; Figure 2*c*). For women, no significant relations between total trauma score and negative symptoms were found (Fig. 2*a*–*c*).

## Discussion

The present study extends previous research on sex differences in the association of childhood trauma with depressive and negative symptoms by investigating a large sample of first-episode psychosis and recent-onset SSD patients, assessing a wide range of childhood trauma subtypes.

Our main findings are the presence of prominent sex differences in trauma prevalence, as women reported emotional abuse rates that are twice as high as men, while sexual abuse was three times as prevalent. In accordance with previous research, we found that childhood trauma is related to more severe depressive and negative symptoms in the total sample (Alameda et al., 2021; Gibson et al., 2016; Stanton et al., 2020; Vargas et al., 2019; Vila-Badia et al., 2021). In contrast to previous research, we found depressive symptoms to be associated with childhood trauma not only in women but also in men. As hypothesized and in line with previous studies, trauma-related associations with negative symptoms were only found in our subset of men and not in women.

In our study, we found differential effects of specific trauma subtypes on depressive symptoms in our total sample and for men and women separately. Both in the total sample and in men, depressive symptoms were associated with the total trauma score and emotional abuse scores. In women, we found a robust association between sexual abuse and the severity of depressive symptoms. Our finding that sexual abuse relates to more depressive symptoms in women but not in men implies that sex differences in the relation between subtypes of childhood trauma and clinical symptoms may contribute to sex differences in the clinical phenotype of SSD, since earlier studies showed more severe depressive symptoms in women than in men with SSD (Fernando et al., 2020; Irving et al., 2021; Quattrone et al., 2018). However, in our study, women did not show statistically significant higher levels of depressive symptoms than men in our study, which was expected as their sexual abuse ratings were three times as high compared to men. Possibly, we did not find any sex differences in the severity of depressive symptoms because the patients included in this study are in remission of psychosis.

Psychological differences between men and women might partly explain the finding that both men and women experience more severe depressive symptoms after childhood trauma, but that childhood trauma affects the severity of negative symptoms only in men and not in women. Some studies show that men are more inclined to show emotional inhibition, resulting in decreased emotional expressiveness, thought suppression, and avoidance (Kring & Gordon, 1998; Matud, 2004), which may lead to depressive and negative symptoms (Degnan, Berry, Humphrey, & Bucci, 2022; Krause, Mendelson, & Lynch, 2003). However, other research suggests that not men but women are more inclined to avoidance behaviour (Panayiotou, Karekla, & Leonidou, 2017). Actually, very few studies investigated the pathways linking childhood trauma and negative symptoms (Alameda et al., 2021). Future studies could investigate sex differences in psychological mechanisms resulting in negative symptoms after a history with childhood trauma.

Our finding that sexual abuse leads to higher levels of depressive symptoms in women is in line with previous findings that sexual abuse in women is associated with high levels of anxiety, guilt, and a pervasive sense of defeat, resulting in a depressed mood (Lysaker & Salyers, 2007; Rhodes, O'Neill, & Nel, 2018). This may be explained by the finding that women are inclined to internalize difficulties, which have been found to result in a higher liability to specifically develop depressive symptoms (Kucharska, 2017; Möller-Leimkühler, 2010; Sigurdardottir, Halldorsdottir, & Bender, 2014).

Furthermore, biological differences are also relevant when interpreting the differential associations between childhood trauma on depressive and negative symptoms in men and women. Differences in response to trauma and stress sensitivity have been found between men and women (De Bellis & Zisk, 2014). Trauma can dysregulate hypothalamus pituitary adrenal (HPA)-axis function, which is thought to result in either lower basal cortisol levels and a dampened HPA-axis response or in a hyperreactive HPA-axis due to sensitization (De Bellis & Zisk, 2014). In men, the first response may be more prevalent, while in women, a hyperactive HPA-axis is more prevalent (De Bellis & Zisk, 2014; Shea, Walsh, MacMillan, & Steiner, 2005). In addition, evidence in rodents suggests that females may be more responsive to the effects of corticotropin-releasing factor (CRF). Indeed, expression of CRF by CRF producing neurons was shown to be higher in females than in males, and sex differences in CRF receptor density, expression, distribution, trafficking, and signalling in certain brain regions might contribute to increased CRF sensitivity in females as well (Bangasser & Wiersielis, 2018). Whether this mechanism is also gender-divergent in human is not yet studied.

Sex differences in the immune system could potentially mediate the effects of trauma on symptom development since immune abnormalities have been linked to both childhood trauma and depressive and negative symptoms. Increased pro-inflammatory cytokine levels including C-reactive protein, Tumour Necrosis Factor-*α*, and interleukin (IL)-6 have been found in SSD patients and healthy participants who experienced trauma (Baumeister, Akhtar, Ciufolini, Pariante, & Mondelli, 2016; Chase et al., 2019). In turn, increased cytokines have been associated with depressive and negative symptoms (Almulla, Al-Rawi, Maes, & Al-Hakeim, 2021; Faugere et al., 2018; Goldsmith & Rapaport, 2020).

So far, no sex effects have been found in relation between childhood trauma and pro-inflammatory cytokines (Baumeister et al., 2016), but sex-specific research is scarce.

The clinical relevance of our results lies in the importance of evaluating trauma history in patients, as this may be linked to a specific symptom profile or presentation. In the treatment of women with SSD and, specifically, those experiencing depressive symptoms, particular attention in trauma therapy should be given to sexual abuse. Indeed, trauma therapy has been shown to result in the experiencing of lower levels of symptoms in SSD patients (Van Den Berg & Van Der Gaag, 2012).

### Strengths and limitations

A strength of this study was the relatively large sample size, evaluating subtypes of childhood trauma and symptoms in 302 first-episode psychosis and recent-onset SSD patients. Moreover, data were corrected for covariates, including age, sex, and antipsychotic dosage. Since we examined a sample with recent-onset SSD (mean illness duration 12 months), long-term antipsychotics use or chronic hospitalisation did not confound our results. This might explain the different findings reported by some previous studies and our current study (Comacchio et al., 2019a; Kelly et al., 2016; Ruby et al., 2017). We do note that the investigated group might not be representative of the first-episode psychosis and recent-onset SSD populations, as patients participating in research are relatively healthy and well-functioning. Larger effects might have been observed in patients experiencing more severe symptoms.

Some studies investigating differences between men and women perform ‘gender-specific’ analyses, while others perform ‘sex-specific’ analyses. ‘Sex’ refers to the biological and physiological characteristics defining males and females, while ‘gender’ refers to the socially constructed roles, activities, and behaviours that a particular society considers to be appropriate for men and women. While it is easy to perform sex-specific analyses, gender-specific analyses demand accurate interviews to assess a person's gender, since gender comprises a spectrum rather than a binary variable such as sex. In our study, we did not interview all participants to assess their perceived gender. Yet, we do know that two participants experienced gender dysphoria who did not undergo gender transition. To complicate things further, their perceived gender at the time of the trauma was different from their currently perceived gender. In this analysis, we classified them according to their sex at birth as we did not have the possibility to investigate gender in our study. We acknowledge that a gender-specific analysis could have provided different findings and is something that should be done as well. From a statistical point of view, we found relatively low rates of childhood trauma (i.e. less than 15% for most subtypes). As a result of this low prevalence, several associations may have been non-significant due to a lack of power. Specifically, this may explain why we did find associations between childhood sexual abuse and depressive symptoms in women but not in men. The sample size of women was relatively small in this study [84 (27.8%)], which might have impacted power as well. Indeed, for some associations, the effect sizes in men are not higher than in women. Therefore, associations between specific types of trauma and negative symptoms in women might be observed in studies using larger sample sizes.

An important strength of our study is that we used the CTQ-SF data as continuous variables in our analyses, rather than as binary variables, in order to account for the severity of the childhood trauma. The reported effect sizes in this study were relatively small, meaning that childhood trauma plays a modest yet significant role in explaining the variance in the severity of depressive and negative symptoms and that other factors also contribute to the symptom severity.

The CTQ-SF questionnaire is a retrospective assessment tool that relies on self-report. Its validity and reliability have been confirmed by earlier studies, showing that results are not affected by current symptoms and that they are consistent over time (Fisher et al., 2011). Recent findings do suggest that the overlap between prospective and retrospective studies may be lower than previously assumed (Baldwin, Reuben, Newbury, & Danese, 2019). However, as sex differences did not explain the differential reporting in prospective and retrospective studies, we do not expect the retrospective nature of the current study to have a significant effect on the observed sex differences in the association of trauma with negative and depressive symptoms.

Moreover, we used the five-factor model of the PANSS to assess depressive symptoms. Although this is a validated scale (Wallwork et al., 2012), the included depressive symptoms are confined to the experience of guilt, depression, and anxiety. We, therefore, did not assess other depressive symptoms such as weight gain or loss, feelings of worthlessness, sleep problems, and concentration problems. This might explain the differences between our findings and the findings of Garcia et al. (2016), Kelly et al. (2016), and Pruessner et al. (2019), who used the Calgary Depression Scale for Schizophrenia (CDSS) and the Brief Psychiatric Rating Scale (BPRS). Furthermore, in our study, anxiety is considered a depressive symptom, while it is investigated as a separate symptom domain in some other studies, possibly influencing the results from sex-specific analyses of depressive symptoms in SSD.

Furthermore, we cannot rule out the possibility that the relation of trauma with depressive or negative symptoms is affected by the difficulty to differentiate between depressive and negative symptoms (Krynicki, Upthegrove, Deakin, & Barnes, 2018).

Finally, in the current study, we did not investigate the association between positive symptoms and childhood trauma. The first reason is that all included patients are in remission of psychosis, resulting in low positive symptom scores. The second reason is that we have chosen to keep the number of statistical analyses limited resulting in a higher power than when performing more statistical analyses.

## Conclusion

Our study found prominent sex differences in the prevalence of sexual and emotional abuse in a large sample of first-episode psychosis and recent-onset SSD patients. As sexual abuse was associated with depressive symptoms only in women, the high rates of depressive symptoms generally observed in women with SSD may partly be explained by their much higher rates of childhood sexual trauma. Total trauma scores as well as emotional abuse and emotional neglect were related to both depressive and negative symptoms in the total patient sample and in men. Lack of power may be a reason why these associations were absent in women. Differential effects of trauma on symptoms across both sexes could be explained by biological and psychological differences between men and women. Given the differential findings in men and women, our results point to the importance of performing sex-specific analyses.

## Supporting information

Enthoven et al. supplementary material Enthoven et al. supplementary material

## Supplementary material

The supplementary material for this article can be found at https://doi.org/10.1017/S0033291723001824.
