# Supplementary material for: The association of childhood trauma with depressive and negative symptoms in recent onset psychosis: a sex-specific analysis
Source: Psychol Med. 2023 Jul 12;53(16):7795–804. doi: 10.1017/S0033291723001824 (PMC10755234; doi:10.1017/S0033291723001824)
Supplement: Enthoven et al. supplementary material [file S0033291723001824sup001.docx]

**Table 1**

*Demographic and Clinical Characteristics for the Simvastatin and the HAMLETT study separately*

|  | Simvastatin study (n = 115) | | HAMLETT study (n = 187) | | P-value Mann-Whitney test | P-value Fisher’s exact test |
| --- | --- | --- | --- | --- | --- | --- |
| Male (n (%)) | 89 (77.4%) | | 129 (69.0%) | |  | 0.073 |
| Age (years) | 27.2 (6.9) | | 28.89 (8.8) | | 0.275 | |
| Illness duration (months) | 15.1 (12.1) | | 10.4 (4.0) | | **0.006**** | |
| Chlorpromazine equivalent dose (mg/day) | 313 (230) | | 220 (139) | | **0.001**** | |
| PANSS |  | |  | |  | |
| Negative symptom subscale PANSS | 14.8 (5.1) | | 11.8 (4.3) | | **<0.001**** | |
| Depressive symptoms (PANSS 5-factor model) | 7.3 (3.0) | | 5.8 (2.5) | | **<0.001**** | |
| CTQ-SF |  | |  | |  | |
| Total trauma score | 41.6 (11.9) | 43.5% | 38.1 (10.7) | 33.2% | **0.003**** | **0.047*** |
| Emotional abuse | 9.4 (4.1) | 21.7% | 7.9 (3.4) | 9.1% | **<0.001**** | **0.002*** |
| Emotional neglect | 12.1 (4.2) | 27.0% | 11.3 (4.3) | 21.4% | 0.084 | 0.166 |
| Physical abuse | 6.4 (2.5) | 7.0% | 5.8 (1.9) | 4.8% | 0.004 | 0.295 |
| Physical neglect | 7.3 (2.6) | 13.9% | 7.1 (2.4) | 11.8% | 0.285 | 0.353 |
| Sexual abuse | 6.4 (3.5) | 14.8% | 6.0 (2.9) | 10.7% | 0.370 | 0.191 |

Data are represented as mean (SD). P-values denote the differences between men and women. For childhood trauma scores, p-values for both continuous measures and prevalence are shown. *p<0.05. **significant after Benjamini-Hochberg FDR correction. The FDR derived significance threshold was 0.027. No FDR-correction was applied for Fisher’s exact test.
